# Innovative antibacterial electrospun nanofibers mats depending on piezoelectric generation

**DOI:** 10.1038/s41598-022-25212-3

**Published:** 2022-12-16

**Authors:** Alaa M. Khalil, Ahmed H. Hassanin, Mai. I. El-kaliuoby, Nada Omran, Mohammed Gamal, Ahmed. M. El-Khatib, Ishac Kandas, Nader Shehata

**Affiliations:** 1 Basic Sciences Department, Faculty of Engineering, Pharos University in Alexandria, Alexandria, 21544 Egypt; 2 Center of Smart Materials, Nanotechnology and Photonics (CSMNP), Smart CI Research Center, Alexandria University, Alexandria, 21544 Egypt; 3 Materials Science and Engineering Department, School of Innovative Design Engineering, Egypt-Japan University of Science and Technology (E-JUST), New Borg El-Arab City, Alexandria, 21934 Egypt; 4 Department of Textile Engineering, Faculty of Engineering, Alexandria University, Alexandria, 21544 Egypt; 5 Physics and Chemistry Department, Faculty of Education, Alexandria University, Alexandria, 21544 Egypt; 6 Physics Department, Faculty of Science, Alexandria University, Alexandria, 21544 Egypt; 7 Department of Engineering Mathematics and Physics, Faculty of Engineering, Alexandria University, Alexandria, 21544 Egypt; 8 Kuwait College of Science and Technology (KCST), 13133 Doha District, Kuwait; 9 USTAR Bioinnovations Center, Faculty of Science, Utah State University, Logan, UT 84341 USA

**Keywords:** Biophysics, Materials science, Nanoscience and technology, Physics

## Abstract

This paper introduces a new approach of testing piezoelectric nanofibers as antibacterial mat. In this work, both Polyvinylidene fluoride (PVDF) and PVDF embedded with thermoplastic polyurethane nanofibers are synthesized as nanofibers mat via electrospinning technique. Then, such mat is analyzed as piezoelectric material to generate electric voltage under different mechanical excitations. Furthermore, morphological and chemical characteristics have been operated to prove the existence of beta sheets piezoelectricity of the synthesized nanofibers mats. Then, the synthesized nanofibers surfaces have been cyclically stretched and exposed to bacteria specimen. It has been noticed that the generated voltage and the corresponding localized electric field positively affect the growth of bacteria and reduces the formation of *K. penomenue* samples bacteria colonies. In addition, the effect of both stretching frequency and pulses numbers have been studied on the bacteria count, growth kinetics, and protein leakage. Our contribution here is to introduce an innovative way of the direct impact of the generated electric field from piezoelectric nanofibers on the reduction of bacteria growth, without depending on traditional anti-bacterial nanoparticles. This work can open a new trend of the usability of piezoelectric nanofibers through masks, filters, and wound curing mats within anti-bacterial biological applications.

## Introduction

The groundbreaking discovery of electrospinning has attracted a lot of attention in both science and business. This mechanism can spin a wide variety of polymers into fibers with diameters ranging from several nanometers to sub-micrometers, along with the control of fibers’ characteristics like pore structure, morphology, and functionality^1,2^. Electrospinning depends on the electric grounding of the collecting target and the stretching electric force produced by the charges delivered to the solution from high voltage power supply. Due to the entanglements in the polymer chain within the fluid, the charged jet does not disintegrate^1^. A droplet at the needle's tip forms a Tylor cone when the charges reach a critical point, and a fluid jet shoots from it in the direction of the grounded collector, which has a lower potential accordingly. Since several polymers whether natural, synthetic, biodegradable, and nondegradable polymers are used to create nanofibers^2^ with unique and creative characteristics including polyurethanes (PU), polylactic acid (PLA), collagen, cellulose, chitosan, silk fibroin, and many more^3–5^, this technique has been used in a wide variety of industries^1^, including pharmaceutical, nano-catalysis, biomedicine, healthcare, protective clothing, environmental engineering, filtration^6^, and many others. Clay, titanium dioxide, carbon nanotubes, silica, and other inorganic nanofillers are used to create nanofiber composites with improved thermal, magnetic, electrical, fluorescent, and catalytic characteristics.

Some polymeric materials such as PVDF have a unique piezoelectric property with the ability to transform the mechanical energy to electrical energy without external inputs^7,8^ as a result of the electric dipole moment of PVDF monomer units due to the β-phase which is responsible for the piezoelectric behavior, and can be enhanced via electric poling and mechanical stretching^9,10^. This property qualifies PVDF to be a promising materials for future manufacturing of several applications, due to the high piezoelectric properties as a result of the polar crystalline structure^11^. The processing of PVDF in the form of nanofibers, play a crucial role in developing a flexible, lightweight, and biocompatible films for several applications such as wearable devices, wound dressing, biosensors^12,13^, and nano-generators^14–16^.

In a correlated track, bioengineering is one of the industries with the fastest growth rates especially when nanofibers technology started to merge inside the field^17,18^. The use of nanofibers in tissue engineering^19^ and wound dressing^20^ to promote cell growth and proliferation^21^ is increasingly crucial as nanofibrous membranes are used in medication delivery systems^22^ to implant drug carriers inside of patients' bodies. By changing the composition, porosity, and shape of electrospun nanofibers, the release rate of a medicine may be controlled while still maintaining an effective drug concentration at the injured area^23^. The electrospun nanofibers, which are excellent transporters, prevent the medication from decomposing. The systemic absorption of the medicine as well as any negative side effects can both be considerably reduced when the nanofibers are utilized as carriers for drug delivery at the specific location of the wound. The theory behind drug delivery using polymer nanofibers is that as the surface area of the drug and the matching carrier increases, so does the rate at which the drug dissolves^24^. The release of a pharmaceutical dosage form can be planned as delayed, immediate, fast, or modified dissolution depending on the kind of polymer carrier used for the creation of nanofibers. Several proteins, polysaccharides, growth hormones, and some anticancer medicines have all been successfully delivered via nanofibers^25–28^. Numerous low-molecular-weight medication solutions have been electrospun by researchers, including lipophilic pharmaceuticals like rifampin, ibuprofen, Cefazolin, Paclitaxel^29^, and hydrophilic drugs including mefoxin and tetracycline hydrochloride^30^.

Strong impact can also be achieved through nanoparticles incorporation inside nanofibers in many applications such as lithography^31,32^, photonics^33–35^, and antibacterial medical applications^36,37^. Strong inhabitation and high antibacterial effect was recorded against several bacteria when using nanoparticles such as silver (Ag)^38^, Zinc oxide (ZnO)^39,40^, and copper oxide (CuO)^41^, which can be used in burns, cuts, and wounds treatment with significant properties^42,43^ for the elimination of bacteria, viruses, fungus^44–46^ and multi-resistant bacteria^47^. Because there is a greater surface area exposed to the germs due to the small fiber diameter, the antibacterial impact is boosted. The nanoparticles in the composite nanofibers begin by rupturing bacterial cell walls, which has the inhibitory effect of preventing bacterial growth and ultimately leads to bacterial death^48–50^. The interaction between the bacterial cell and the oxides is facilitated by the nanoparticles' tiny size^51^. The method of nanoparticle penetration into bacterial cells is described by osmosis, cell-destructing enzymes, and subsequent cell death.

Along with the interest in finding alternatives to antibiotics, great attention has arisen to studying the effects of electromagnetic waves and their ability to control bacterial growth as an alternative technique rather than the use of antibiotics^52–54^. It has been proven that the electric field has a role in inactivating microorganisms under non-thermal conditions^55–57^. There are several parameters affecting the role of field interaction such as bacteria type, growing phase, and conditions of growth. Not only this but also, the field type, strength, frequency, time of application, and field proximity to exposed cells^58–60^. The metabolic process of bacterial cells possesses various intercommunication signals that acting between cells to complete the vitality process^61^. The application of external localized fields may cause disruption of cellular membrane charge distribution and hence lead to electrical stress on the bacterial bio-entity^55,62–64^. Even at very low energy electric fields, they have the ability to penetrate the biological systems with almost no attenuation and to modify the natural biorhythms and existing signal transduction processes in cell membranes^65^. The transmembrane receptors and vertebral macromolecules allocated through the cellular envelop are acting as a group of electric fields antennae that can differentiate, discern, and transform the wave energies into signals^66^. Consequently, the impact application of close proximity electric fields may lead to severe cellular structural changes that eventually lead to lethal effect and drive to cell lysis^67^.

In this work, we are introducing an innovative antibacterial impact of piezoelectric nanofibers to use the generated localized electric field from mechanical excitations, such as cyclic tension, into a direct effect on bacteria. The used nanofibers are composed of pure PVDF nanofibers and PVDF mixed with Thermoplastic polyurethane (TPU) to give more mechanical stretching capabilities. From a recent published work by the authors, the addition of TPU at certain optimum concentration can lead to enhance the piezoelectric sensitivity due to the higher mechanical stretchability that TPU can contribute^68^. Then, different piezoelectric analysis has been operated to show the generated electric voltage under different mechanical excitations including cyclic tension, cyclic normal force, and impulse loading. In addition, beta phase analysis using both XRD and FTIR is carried out to prove the piezoelectric performance of the synthesized nanofibers mats. Therefore, the stretched piezoelectric nanofibers are exposed to a specimen of bacteria to check the effect of generated voltage and corresponding localized electric field on the life of the bacteria colonies and its kinetic growth. Different stretching frequencies and number of stretching cycles are examined on the antibacterial performance.

## Materials and methods

### Materials

Polyvinylidene fluoride (PVDF) was purchased from ARKEMA (Kynar®, King of Prussia, PA, USA), Thermoplastic polyurethane (TPU) was ordered from (BASF Co., Ltd., Berlin, Germany) with 107,020 g mol^−1^ molecular weight and Polydispersity Index (PDI) of 1.83. Certain concentrations of the polymers were dispersed in dimethylformamide (DMF 98%) from (Sigma Aldrich, Taufkirchen, Germany).

### Membrane fabrication

PVDF polymer solution of 10 wt% was prepared by adding the polymer powder into DMF. Similar concentration of TPU polymer solution was prepared by dispersing the polymer pellets into DMF, and a blended ratio was prepared with total concentration of 10% for PVDF:TPU, but with a weight ratio equals to 85:15, respectively. Knowingly that, this ratio has been chosen according to previous study performed by the same research team (68). The prepared polymer solutions were stirred overnight before the spinning process. Electrospinning process was performed by adding the prepared polymer solutions of PVDF and PVDF/TPU into plastic syringes with stainless steel needles of gauge 21. A high voltage power supply provided 30 kV (CZE1000R, Spellman, Hauppauge, NY, USA) to the syringe needle, and a constant feed rate of 1 mL/hr was fixed by NE1000 syringe pump (New Era Pump Systems, Suffolk County, NY, USA). A distance of 10 cm was adjusted between the needle and the grounded drum collector.

### Morphological, Chemical and Mechanical Characterizations

Scanning Electron Microscopy through (JEOL JSM-6010LV-SEM, Tokyo, Japan) was performed to observe the surface morphology of PVDF and PVDF/TPU nanofibrous membranes with 15 kV acceleration voltage. The nanofiber membranes were fixed on a carbon tape over aluminum stubs and sputter coated with platinum. The nanofibers diameters were analyzed through Image-J software (Madison, WI, USA) and the fiber diameter distribution was detected manually at different imaging scales. The Fourier Transform Infra-Red Spectrometer (FT-IR) (Vertex 70 FT-IR, Bruker, Billerica, MA, USA) was used to calculate the β phase content in ATR mode. The nanofibers were scanned 120 times at a resolution of 5 cm^−1^ over a range of 4000–400 cm^−1^. The mechanical tensile testing was performed through Texture Analyzer CTX (AMETEK Brookfield, Middleboro, USA) by cutting the produced nanofibrous membranes into rectangular (1 × 6 cm^2^) and placing each sample between two cardboards frames. The thickness was measured by spring-based micrometer. Strain rate of 10 mm/min and zero initial loads were fixed. The used load cell was equal to 50 N. Each sample was tested three times and then the average stress–strain curve has been analyzed, to detect maximum tensile strength and the breaking strain where the strain where the sample was cut.

### Piezoelectric Analysis

**Figure 1:**
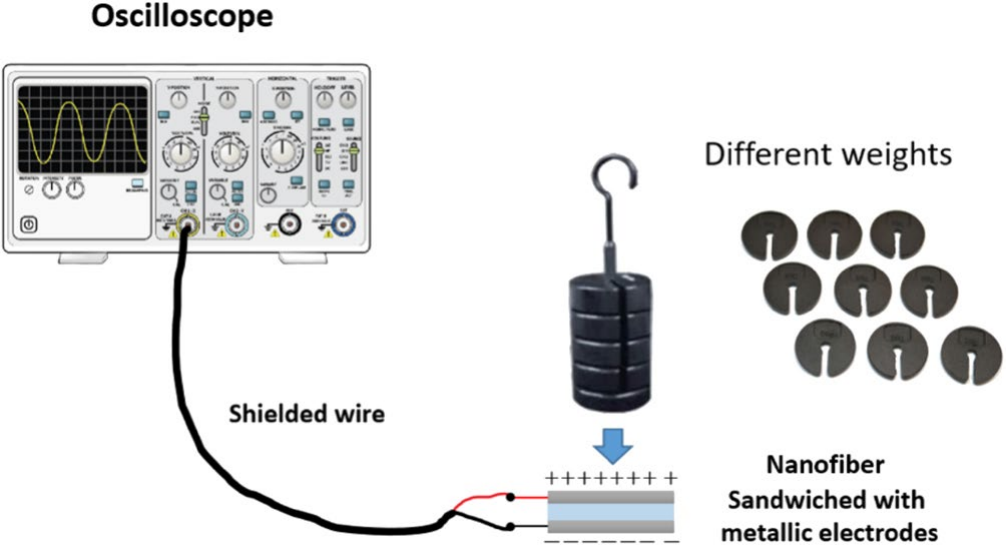
The schematic of the impulse loading setup with different weights^68^.

The Piezoelectric characterizations for pure PVDF and PVDF: TPU 15% were measured using various testing such as impulse, frequency, and stretching responses. The impulse response has measured using a simple setup as shown in Fig. 1. The nanofiber samples were sandwiched between two conductive materials connected with a high impedance oscilloscope (Tektronix MDO3014), using isolated wire. A different weights 20–150 g have been thrown from 1 cm height onto the nanofiber system and the output voltages have been measured using the oscilloscope.

**Figure 2:**
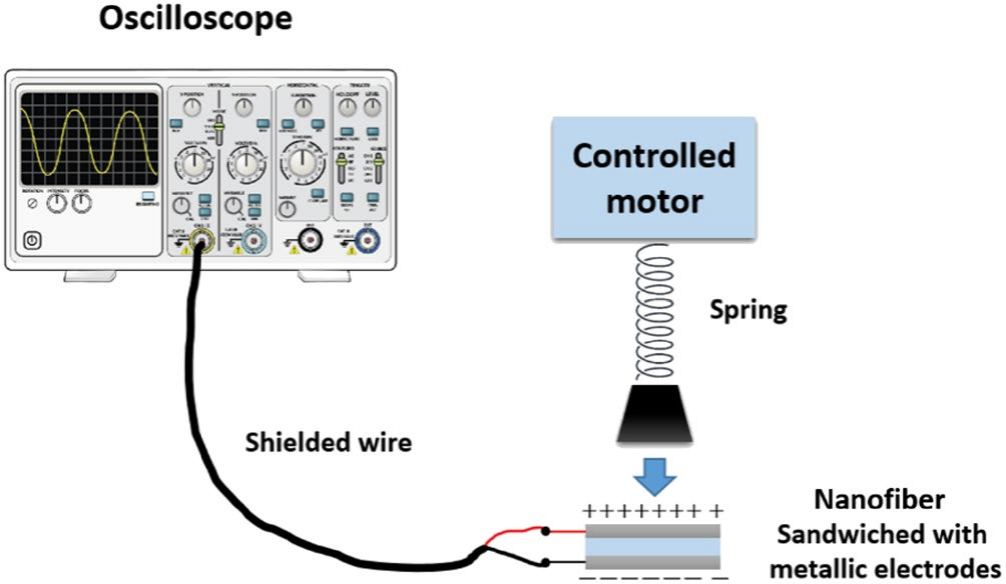
The cyclic piezoelectric schematic^68^.

**Figure 3:**
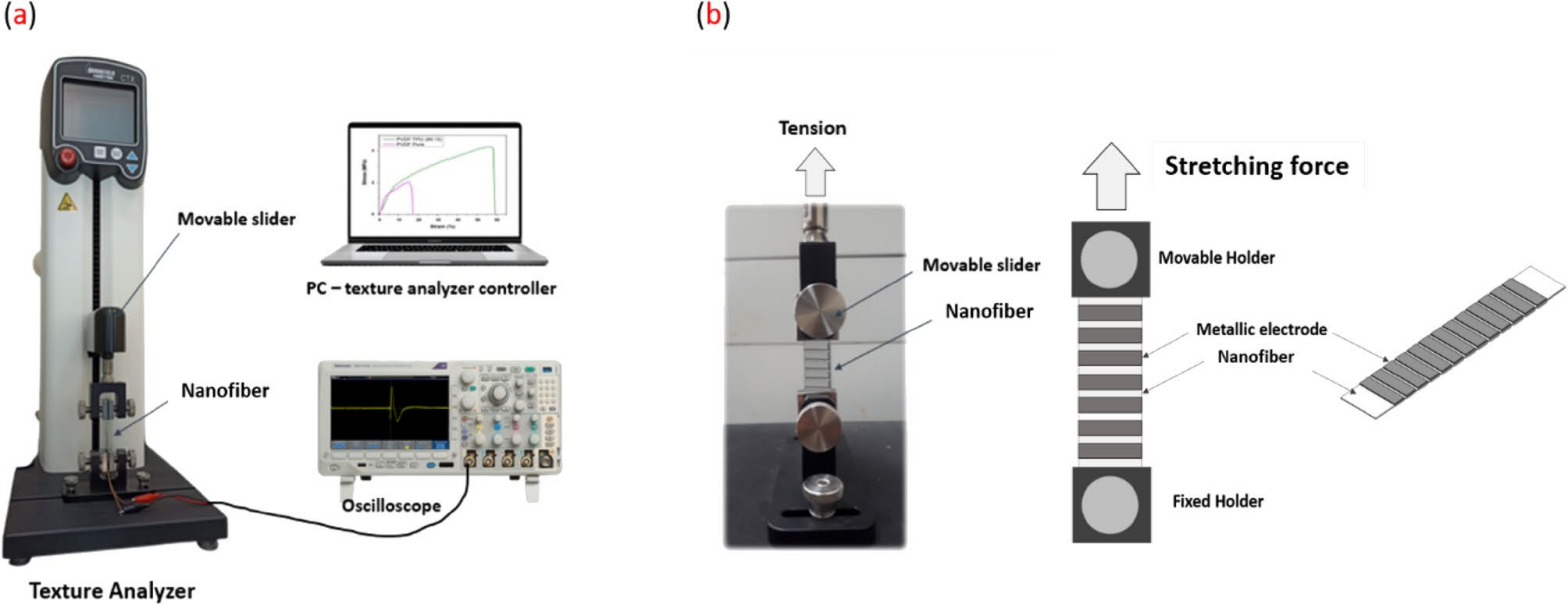
(**a**) The stretching piezoelectric setup and (**b**) the sandwiched sample between two stretchable metallic electrodes.

In another test, the cyclic load has been applied on nanofiber system for measuring the frequency response using a light-weight spring built-up vertically controlled by brash-less DC motor connected with an electronic speed controller as shown in Fig. 2. The cyclic load pressed on the sandwiched nanofiber with applied range force 1–4 N, and the peak-to-peak output voltage was measured by the oscilloscope. For piezo-analysis under stretching impact, the piezoelectric stretching test is controlled by Texture Analyzer with frequency 1 Hz as shown in Fig. 3a, the sample dimensions is 1 × 4 cm^2^ and sandwiched between two stretchable metallic electrodes as shown in Fig. 3b. The electrodes are wired together and the output voltage is measured with the oscilloscope.

### Antibacterial Characterization

Standard strain of *K. pneumoniae* (ATCC 70068) was collected from Alexandria University Hospital, Egypt. It is worthwhile clarifying that the chosen bacteria strain is based on recent findings in our lab which confirmed the antibacterial effect of exposure to electromagnetic fields as a stressor co-factor against *K. pneumoniae*^69,70^.The isolates were inoculated in MacConkey agar plates at 37 °C for 24 h. For maintaining fresh subcultures, every while three colonies were added into sterilized MacConkey broth media and incubated at 37 °C for 24 h. Then after, a cultured supernatant stock inoculated in MacConkey broth by approximately 10^5^ CFU of *K. pneumonia* was prepared. Four groups of bacteria samples are collected after exposure to electric pulses generated from nanofibers at three different exposure conditions and one group of bacteria sample free of exposure is considered as a control one. It is worthy to state that the nanofiber sheets were stretched mechanically in three frequency bases (0.5, 1.0, 1.5, 2.0 Hz) and every time the bacteria samples were sprayed over the sheets before stretching and collected by taking swaps after the mechanical stretches. The growth characteristics of bacteria samples were evaluated by measuring growing turbidity in broth media and plate counts in agar media. The optical density (OD) of turbid inoculated broths were monitored every 1 h by taking 1 ml from 25 ml transparent glass bottles of the supernatants under treatment in a semi-micro quartz cuvette (P.N. 035 127). The OD measurements were carried out by using a spectrophotometer (Jenway, 6405 UV/vis, Essex, UK) set at 600 nm and every 0.1 in OD reading scale is considered as 10^8^ bacteria cells per cm^3^^71^. Furthermore, the growth counts were measured by using plate counting technique to get CFU values confirming the obtained OD measurements. The growth curves between OD values and incubation times were graphed. The curves were analyzed and corresponding arbitrary rate constants were calculated according to the following formulae and graphed versus concentrations for each applied frequency^72^.

$${\text{Arbitrary}}\,{\text{growth}}\,{\text{rate}}\,{\text{constant = }}\left( \frac{1}{t} \right)\ln \left( {\frac{N}{{N_{o} }}} \right),$$

where N is the bacterial cell count at the time (t) and N_o_ the initial cell count. In addition, the cytotoxicity was assessed by lactate dehydrogenase (LDH), nucleic acid and protein leakage into the culture medium. The levels of LDH values, protein leakage amounts and nucleic acid percentages were obtained by methods adapted by Kim et al*.*^73^, Li et al*.*^74^, and Riss et al*.*^75^, respectively. Results were analyzed and presented as a percentage of control values and graphed relative to it.

## Results and Discussion

### Morphological Characterization

**Figure 4:**
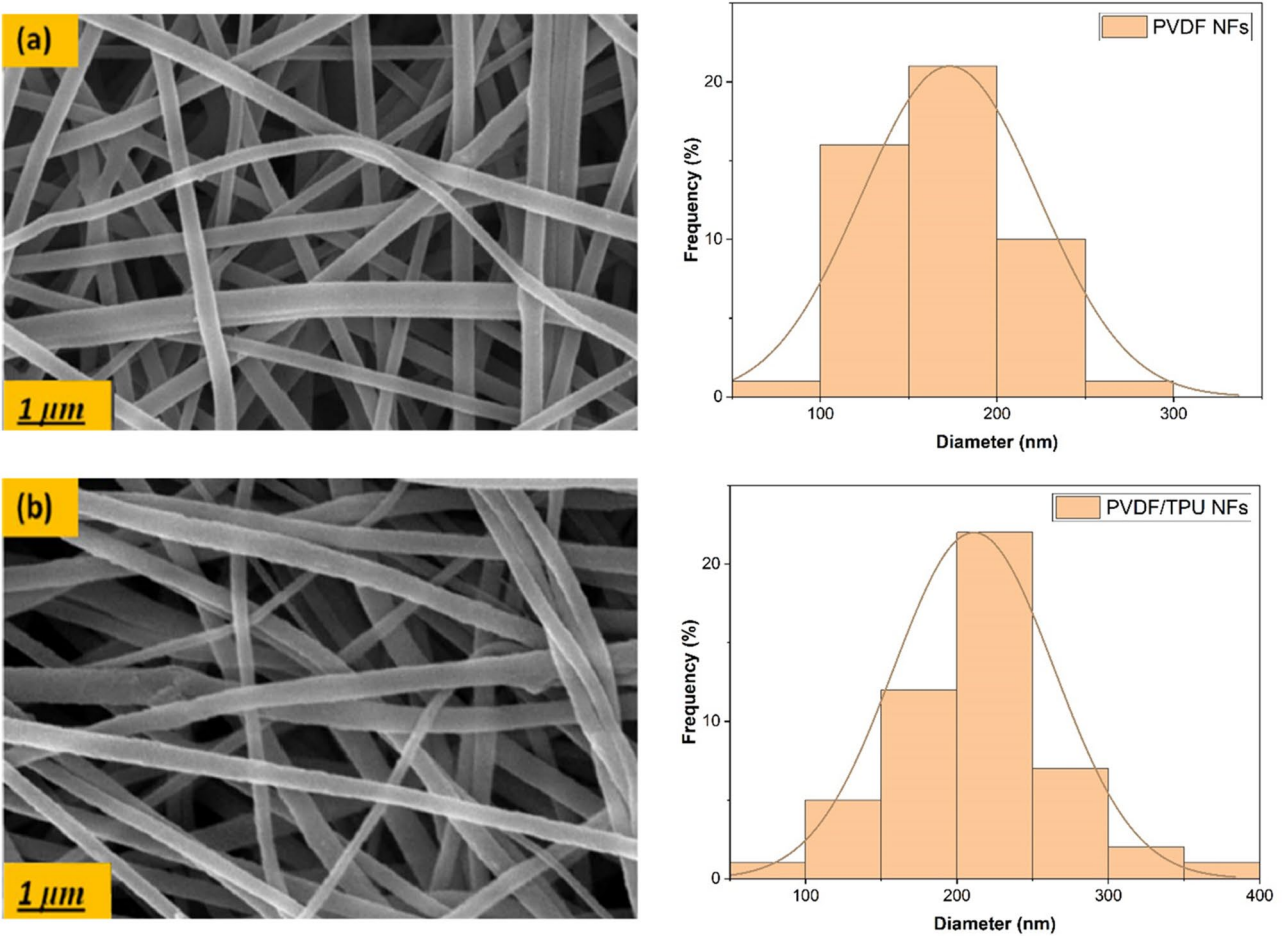
SEM images and fiber diameter distribution of (**a**) PVDF; (**b**) PVDF:TPU (85:15) NFs membranes.

Figure 4 shows SEM images of the produced PVDF and PVDF/TPU (85:15) nanofibrous membranes which clarifies homogeneous and smooth surface morphology with minimized beads or agglomerations. Then, TPU was smoothly well-mixed within the PVDF solution and that mixture generally forms an excellent polymer chain entanglements with nearly no formed beads in the stage of nanofibers formation. The average fiber diameter and fiber distribution was calculated, as TPU addition increases the diameter of the nanocomposite with achieving mean fiber diameter of 173 nm and 211 nm for both pure PVDF and PVDF/TPU (85:15), respectively, proving the high compatibility and blending of the polymer solutions. The reason of the diameter’s increase is mainly coming from both reduced electrolyte property and dielectric nature of TPU. Therefore, when TPU was affiliated, less ions were formed in the blend solution with reduced charge density could be carried by the electrospinning jet and consequently less solution conductivity. That will lead to less stretching of the electrospun jet and then larger formed mean diameter of PVDF:TPU nanofibers, compared to pure PVDF^76,77^.

### Physical Characterization

**Figure 5:**
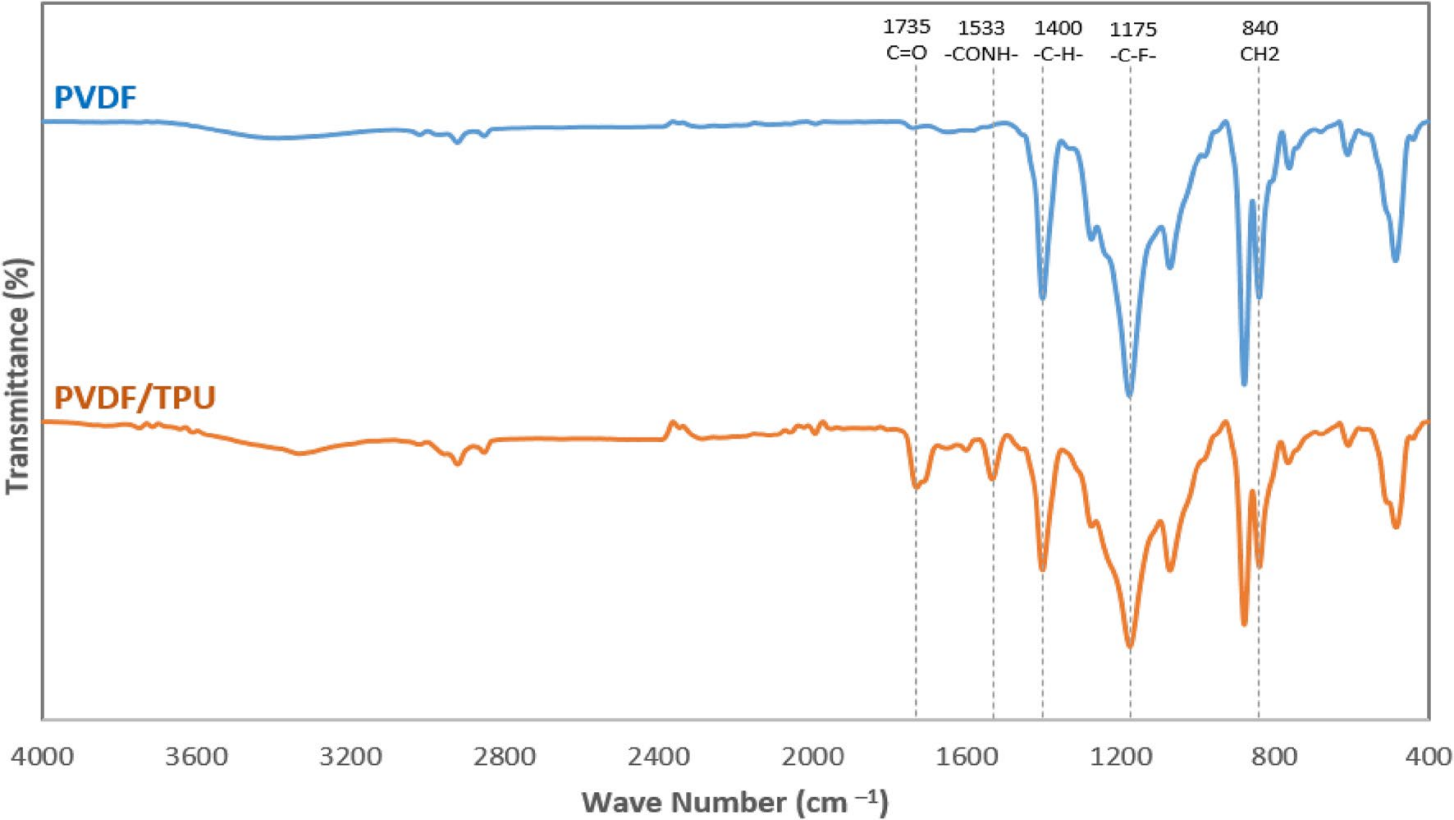
FT-IR analysis of PVDF and PVDF/TPU (85:15) nanofibers.

Figure 5 shows the FT-IR spectra for the produced nanofibrous membranes to study the crystalline phases for the PVDF nanofibers. Since the β-phase is the responsible for the enhancement of the piezoelectric property, increasing the β-phase content improves the piezoelectric response in the produced nanofibers. It was observed that electrospinning process has a positive impact on the piezoelectricity of the PVDF due to the high electric field effect on aligning the dipoles^78^. As shown in Fig. 5, the characteristic bands of PVDF appeared at 840 cm^−1^ corresponding to the rocking of CH_2_, C–C, and CF_2_ stretching, in addition to 1175 cm^−1^ for C–F and 1400 cm^−1^ for C–H vibrations^78–80^. Nevertheless, the main characteristic bands for TPU were observed at 3365, 2971, 1735, and 1533 cm^−1^, corresponding to N–H stretching, C–H, C = O, and CONH– asymmetrical bond, respectively^81,82^. The β-phase fraction was calculated as shown in Table 1, through the following equation:

$$F\left( \beta \right) = \frac{{A_{\beta } }}{{1.26 A_{\alpha } + A_{\beta } }}$$

**Table 1:** Calculated β-phase Fraction for PVDF and PVDF/TPU nanofibers^68^.

Sample	A_α_	A_β_	F(β) content (%)
Pure PVDF	0.029	0.138	79.06
PVDF:TPU (85:15)	0.015	0.076	80.00

where *A*_*α*_ and *A*_*β*_ are the intensities of 764 cm^−1^ and 840 cm^−1^ absorbance bands, respectively. Table 1 shows that the addition of TPU to PVDF enhances the formed β-sheets concentrations inside the nanofibers. Although the concentrations of both *A*_*α*_ and *A*_*β*_ were reduced according to the addition of TPU, the β-phase fraction was increased within PVDF: TPU sample compared to pure PVDF membrane. Accordingly, the piezoelectric response can be developed for TPU added samples compared to the pure PVDF ones, as will be shown in a later section.

### Mechanical Characterization

**Figure 6:**
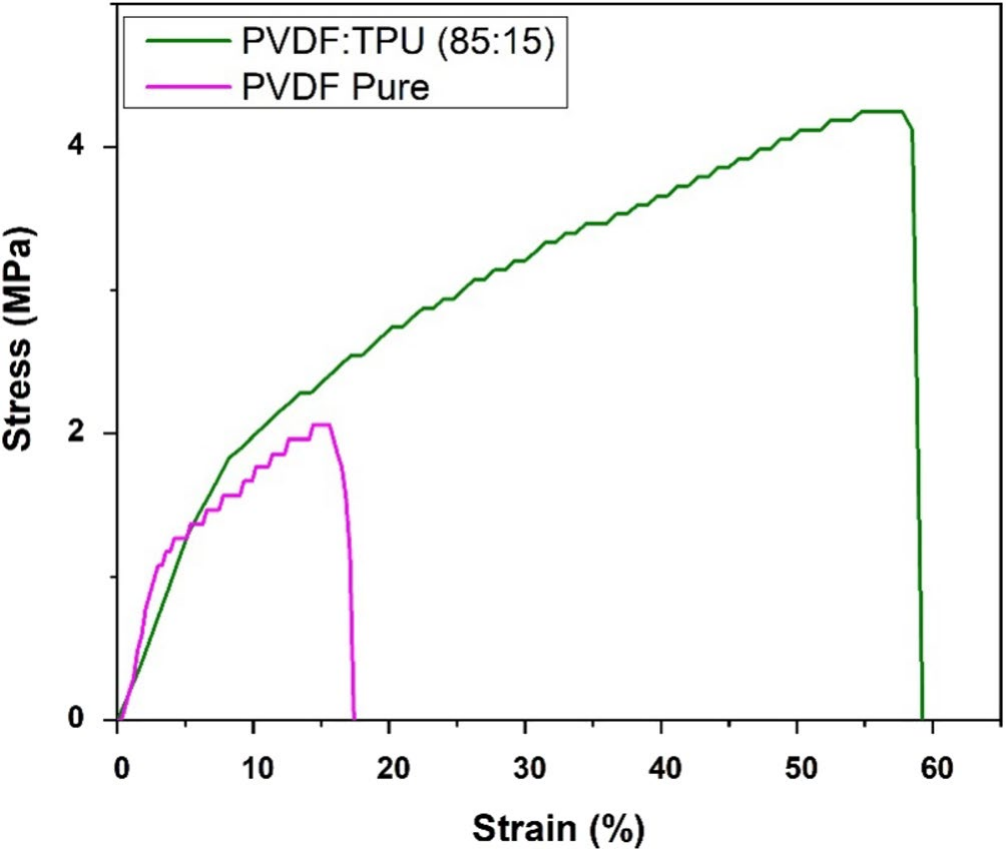
Stress–strain curve for PVDF and PVDF/TPU NFs membranes.

Stress–strain curves of PVDF and PVDF/TPU nanofibrous membranes are shown in Fig. 6. Significant improvement in the mechanical properties was observed by the addition of TPU to the polymer matrix. Pure PVDF nanofibrous membrane achieved tensile strength of 2.1 MPa and breaking strain equal to 17%, while PVDF/TPU showed higher elasticity with tensile strength of 4.2 MPa and elongation at breakage of 59%. Adding TPU enhanced the mechanical properties making the composite membrane a promising candidate for different applications according to the enhanced mechanical stretchability^83–85^.

### Piezoelectric Analysis

**Figure 7:**
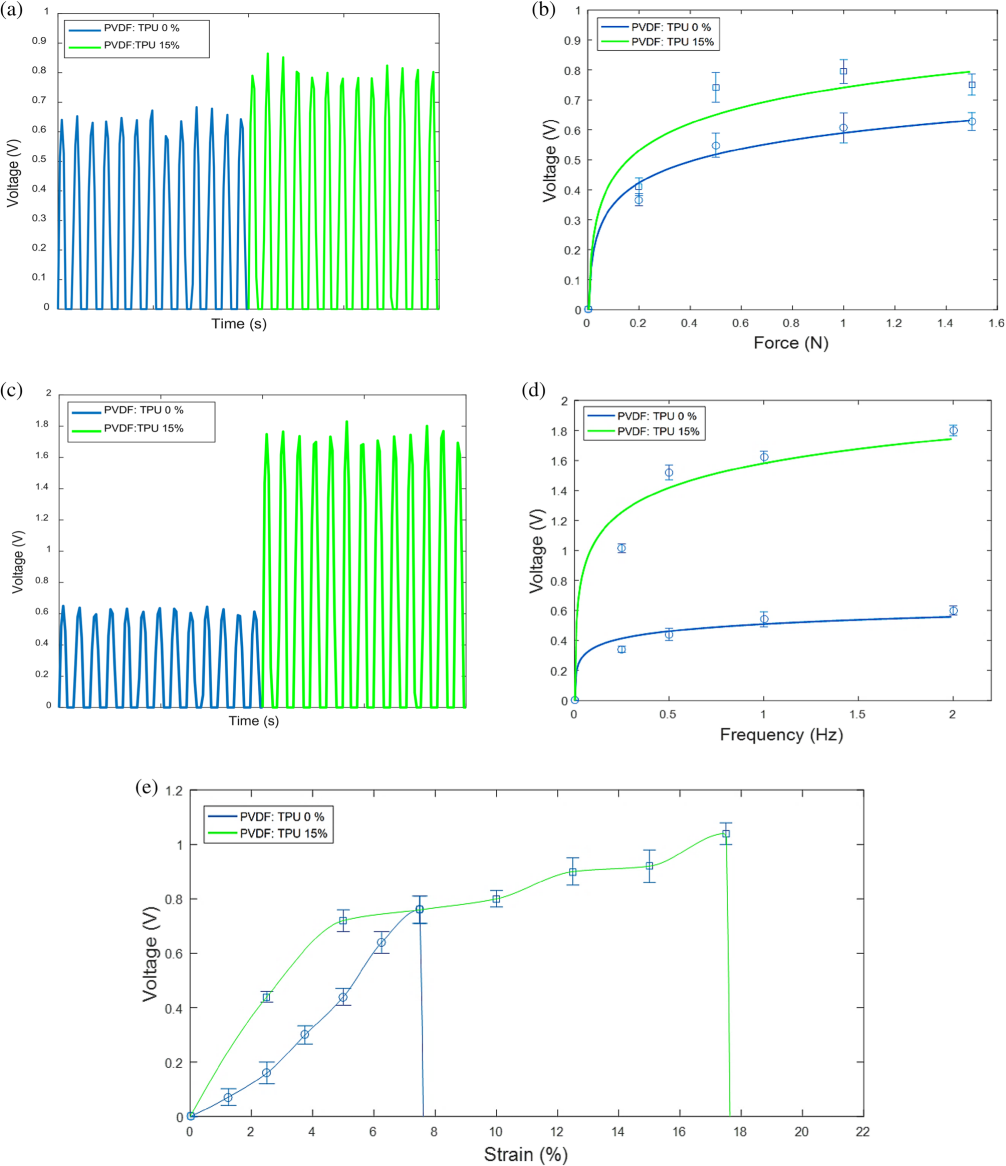
The results of the piezoelectric characterization under (**a**) the output voltages with time at 1.5 N, (**b**) the impulse response with different applied forces, (**c**) The output voltages with time at 1.5 N and 2 Hz, (**d**) The frequency response at constant force 1.5 N, and (**e**) the stretching response.

In this section, the generated peak-to-peak output voltages are detected under different applied mechanical excitations including impulse loading of free fallen masses with different weights, mechanical cyclic forces, and cyclic stretching/tension. Figure 7 shows that as the impulse weight, frequency, and the stretch increase the output voltage increase and tend to be saturated which gives us an indication to the maximum material polarization. For the force/voltage test, the output voltage curve of PVDF: TPU 15% nanofiber starts to be saturated at 0.8 V when force of 1.5 N is applied, whereas the pure PVDF nanofiber saturated at 0.6 V as shown in Fig. 7b. Now, when a fixed pressure force of 1.5 N at different cyclic frequencies is applied, the saturated curve of PVDF: TPU 15% get enhanced to 1.75 V at 2 Hz as shown in Fig. 7d. Figure 7e shows the piezoelectric sensitivity of the mat at different stretched strains. It is noticed that the results of PVDF: TPU 15% mat is quite larger than the pure PVDF mat up to ~ 8% strain limit. Beyond this limit, the PVDF: TPU mat is able to be stretchable and generate larger voltage, while the pure PVDF nanofibers reached to the breaking point.

From the previous discussion, the output peak-to-peak voltage of the blended PVDF: TPU mat is found to be larger than the pure PVDF for the impulse, cyclic, and stretching responses. In addition, the comparison between the impulse and frequency responses shows that the response of blended PVDF:TPU, according to the frequency increase is more effective than the incremented applied force as shown in both Fig. 7a,c. Also, the blended PVDF:TPU has better stretching property, which gives us an obvious improvement in the piezoelectric output voltage according to its better face-shear piezoelectricity which is related to the increased possibility of electric dipoles alignment due to the stretching nature of added TPU^68,86,87^.

### Antibacterial Characterization

Now, it will be shown the effect of exposing gram-negative bacteria *Klebsiella penomenue* (*k. penomenue*) to piezoelectric field pulses (PEPs) generated from PVDF and PVDF-TPU nanofibers under application of mechanical stretches. The antibacterial effect of PEPs was obtained by measuring the growth kinetics over 20 h of incubation and by plate colony counts. Furthermore, bacterial cytotoxicity was investigated by measuring the percentage of protein leakage, the level of the lactate dehydrogenase enzyme (LDH), and nucleic acid changes in comparison to control samples. It is worth to note that the PEPs are generated from mechanical stresses applied over the PVDF nanofiber mats, and so, here we divided the groups of nanofiber samples based on the number of applied mechanical stretches. The applied mechanical stretches were adapted by time of application and represented as frequency “in terms of Hz” resulted from dividing different number of pulses per relevant sets of times (*f* = 0.5, 1, 1.5, and 2 Hz). It is worthwhile clarifying that the generation of PEPs was done by applying different numbers of mechanical stretches within certain periods at the aforementioned frequencies to check the influence of pulse numbers and stretching times as an antibacterial agent. The bacterial log reduction values for *k. penomenue* samples treated by PEPs generated from PVDF and PVDF-TPU nanofibers at set of frequencies are tabulated in Table 2. The obtained log reduction values showed remarkable difference of samples treated by PEPs under application of PEPs in frequency and nanofiber type dependent manner. Significantly, the maximum bacterial reduction by 4.7 log (99.99% reduction) was obtained at frequency 2.0 Hz due to PEPs generated from PVDF-TPU nanofibers. Besides that, the effect of applied mechanical stretches on PVDF-TPU nanofiber mats remarkably influenced the growth of bacteria count in a way that as many stretches were applied, or in another way the generated pulses, hence a higher bacteriostatic effect was obtained.

**Table 2:** Growth bacteria counts and log reduction (relative to control count 950 × 10^10^ CFU/ml) under effect of PEPs generated from PVDF and PVDF-TPU nanofibers at set of frequencies (f = 0.5, 1, 1.5, and 2 Hz).

Frequency *f,* Hz	Number of pulses	Stretching time, s	Nanofiber sample	Bacteria count	Log reduction
0.5	15	30	PVDF	950 × 10^10^	0.035
	15	30	PVDF-TPU	760 × 10^10^	0.132
1.0	30	30	PVDF	101 × 10^9^	2.009
	30	30	PVDF-TPU	910 × 10^8^	2.054
1.5	30	20	PVDF	860 × 10^8^	2.078
	45	30	PVDF-TPU	800 × 10^7^	3.110
2.0	30	15	PVDF	606 × 10^8^	2.412
	60	30	PVDF-TPU	191 × 10^6^	4.732

**Figure 8:**
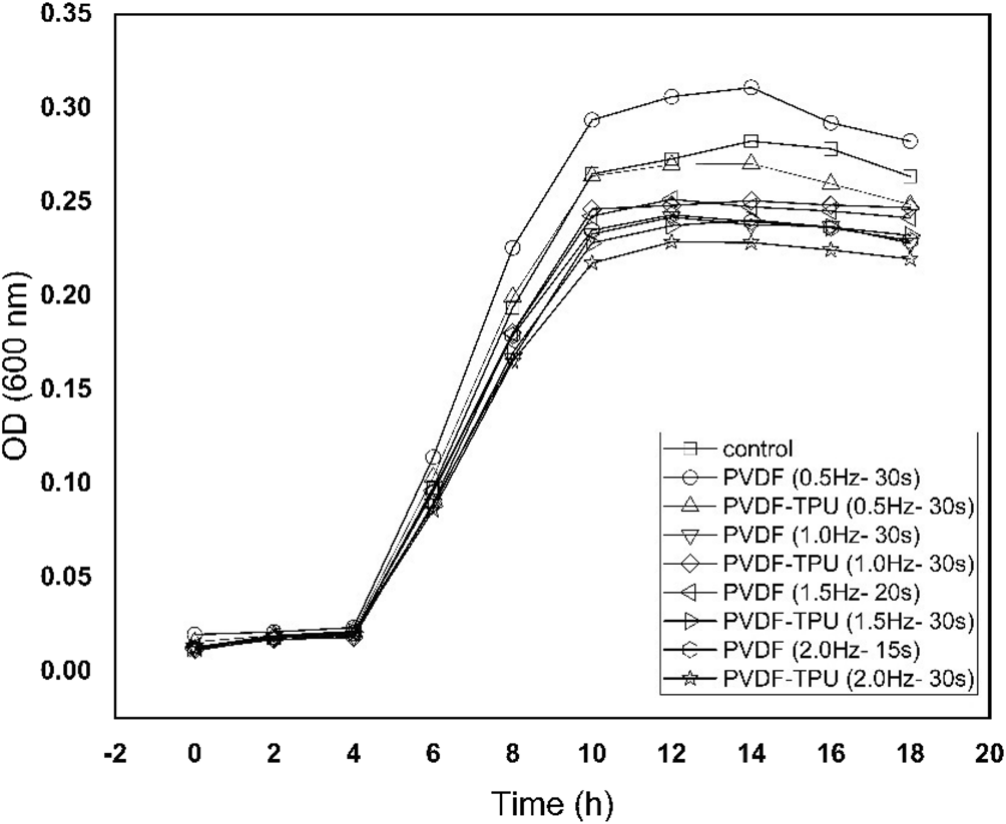
The growth kinetics of k. penomenue samples exposed to PEPs generated from PVDF and PVDF-TPU nanofibers at set of frequencies (f = 0.5, 1, 1.5, and 2 Hz).

**Figure 9:**
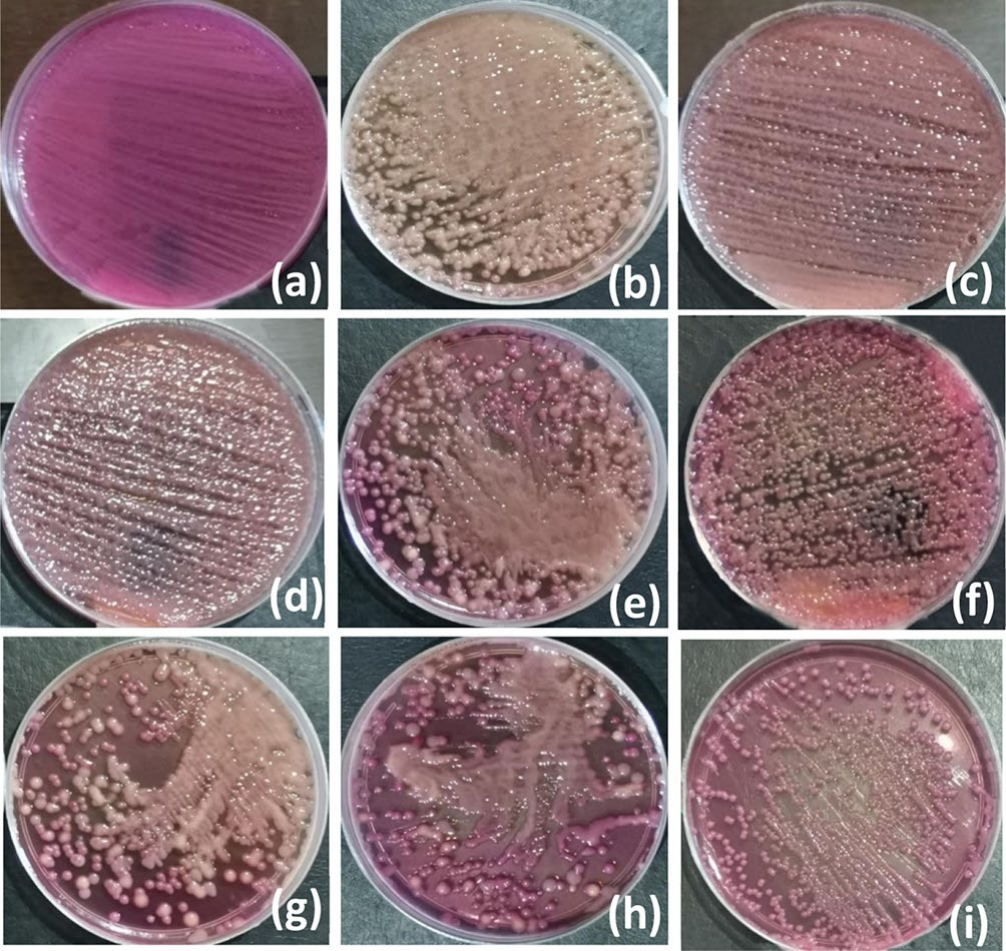
The petri dishes images of k. penomenue colonies cultured in MacConkey media for control samples − 0.0 Hz (**a**), and samples exposed to PEPs generated from PVDF at 0.5 Hz-30 s (**b**), 1.0 Hz-30 s (**c**), 1.5 Hz-20 s (**d**), 2.0 Hz-15 s (**e**) and from PVDF-TPU at 0.5 Hz-30 s (**f**),1.0 Hz-30 s (**g**), 1.5 Hz-30 s (**h**), 2.0 Hz-30 s (**i**).

**Figure 10:**
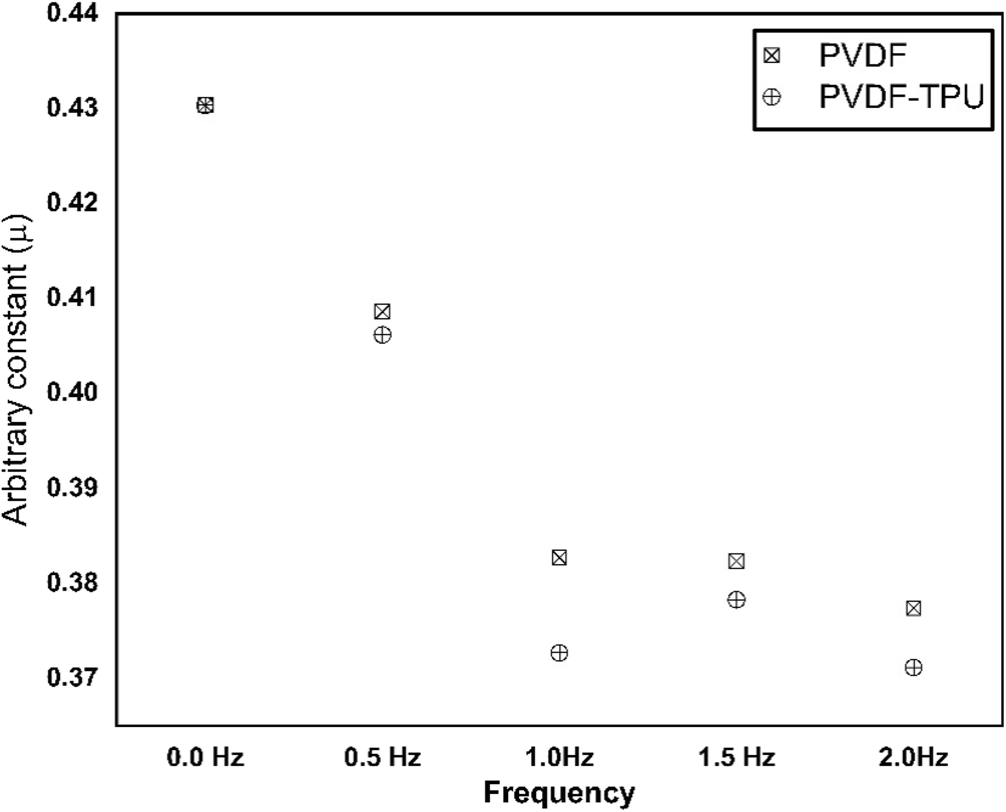
The growth arbitrary constant values of k. penomenue samples exposed to PEPs generated from PVDF and PVDF-TPU nanofibers at set of frequencies (f = 0.5, 1, 1.5, and 2 Hz).

Furthermore, bacteria growing kinetics were studied and graphed under the application of PEPs generated from PVDF and PVDF-TPU nanofibers at set of frequencies (f = 0.5, 1, 1.5, and 2 Hz) as shown in Fig. 8. The images of *k. penomenue* colonies grown in MacConkey media exposed to PEPs generated from PVDF and PVDF-TPU nanofibers at set of frequencies (f = 0.5, 1, 1.5, and 2 Hz) in comparison to control one are represented in Fig. 9. It is worthy to mention here that the images were shown for presentation purpose to clarify the difference in growing density under treatment conditions; while the countable petri dishes were adopted to the streak plate method at serial dilutions. The measurement of optical density (OD) represents the density of bacteria growth in accordance to its count and could be taken to demonstrate the inhibition characteristics. In addition, the growth curves were mathematically analyzed and its corresponding arbitrary growth constants for each curve were calculated. The arbitrary constant values versus frequencies for PVDF and PVDF-TPU nanofibers were graphed as shown in Fig. 10. The characteristic growing curves in Fig. 8 illuminated normal growth phases without any abnormalities for all treated samples as compared with control ones. Also, the maximum growth depression was shown for bacteria samples treated by PEPs generated from PVDF-TPU nanofibers at 2 Hz. The arbitrary constant is an index of the growth rate and reflects the possible changes in bacterial growing kinetics as a result of treatment by PEPs. Hence, the sequential decrease in the constant values is shown in Fig. 10, which points to a higher antibacterial effect resulted from PEPs generated from PVDF-TPU nanofibers through the frequencies range (1, 1.5, and 2 Hz). The obtained ultimate growth inhibition was at frequency 2 Hz for both PVDF and PVDF-TPU nanofibers.

**Figure 11:**
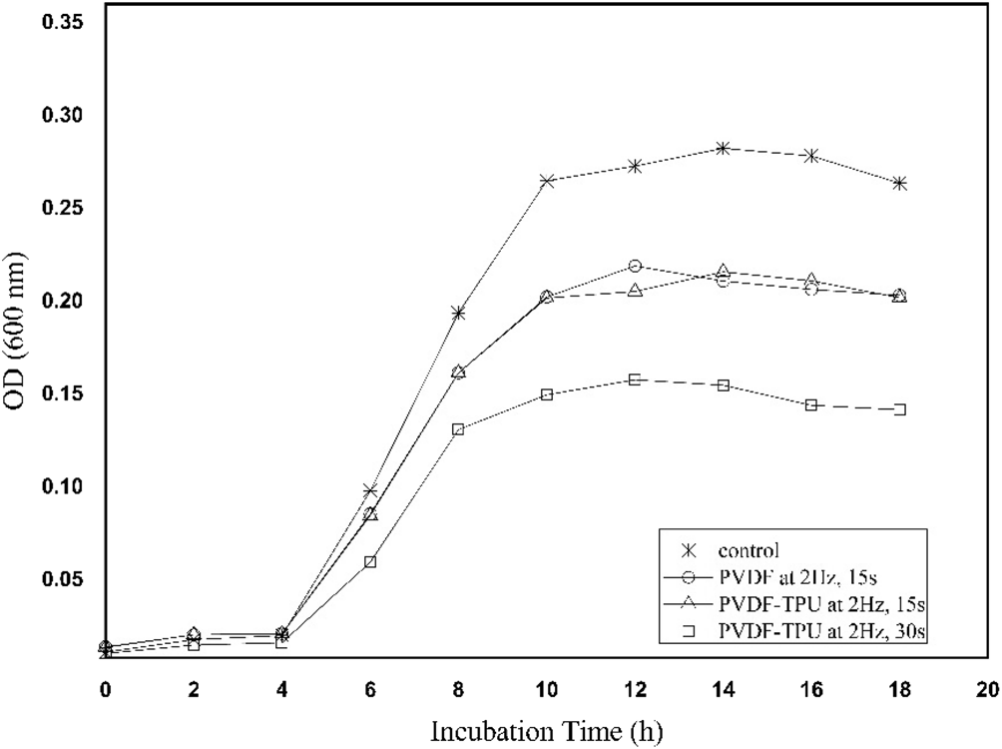
The growth kinetics of k. penomenue samples exposed to PVDF 2.0 Hz (15 s) and PVDF-TPU 2.0 Hz (15 and 30 s) nanofibers.

To that end, the growing kinetics of bacteria exposed to PEPs, which were generated from nanofibers mats, were graphed together to compare the effect of time and the number of applied mechanical stretches as shown in Fig. 11. It is worthwhile clarifying that because of the lower elasticity of PVDF than PVDF-TPU samples, it wasn’t able to make mechanical stretches more than 30 pulses. Therefore, for PVDF samples, the frequency of 2 Hz was adapted only to 30 pulses over 15 s and for PVDF-TPU samples it was adapted to 30 and 60 pulses over 15 and 30 s respectively. The growth characteristics in Fig. 11 indicate that the inhibition due to PEPs generated from PVDF and PVDF-TPU of 30 pulses are typically similar. Moreover, the influence of pulse number on the inhibitory effect indicated a significant increase by 70% of PVDF-TPU at 60 pulses compared to 30 pulses. Therefore, an expressively exposure to localized fields generated from nanofibers showed remarkable influence on bacteria' cells vitality. The pulse number has the leverage action against bacterial bioactivity in resemblance to a frequency-dependent manner^86,87^. It is worth to note that live cells possess intercommunication signals between each other to functionalize their metabolic activities^61^. The transmembrane specialized receptors and allocated protein molecules, acting as a group of electromagnetic antennas that can differentiate, discern, and transform the wave energies into signals^88^. In another way, the external electromagnetic fields have the ability to penetrate the biological systems without attenuation and they have the ability to modify the natural bio-rhythm even at very low energies^89^. Therefore, the train of applied pulses from PEPs generated from nanofibers has influenced the existing signal transduction processes in cell membranes by 0–1 field action because of its analogy to frequency resonance action^90^.

**Figure 12:**
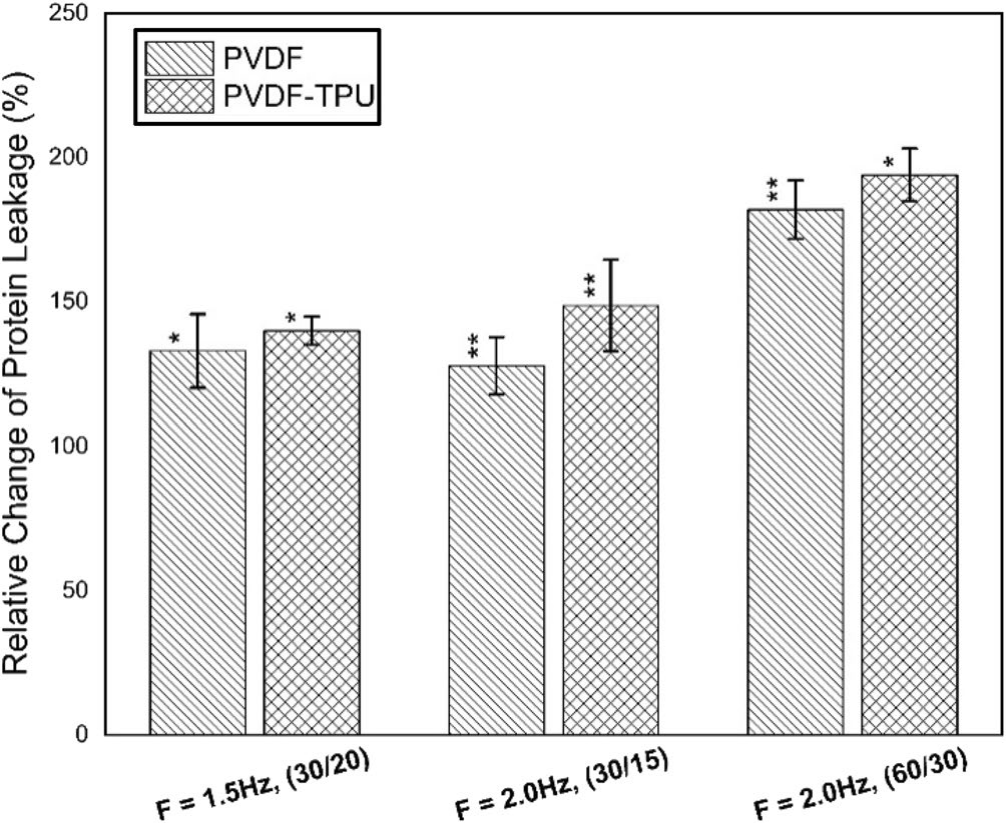
The relative protein leakage percentages of k. penomenue samples exposed to PEFPs generated from PVDF and PVDF-TPU nanofibers at 1.5 Hz-20 s, 2.0 Hz-15 s and 2.0 Hz-30 s.

**Figure 13:**
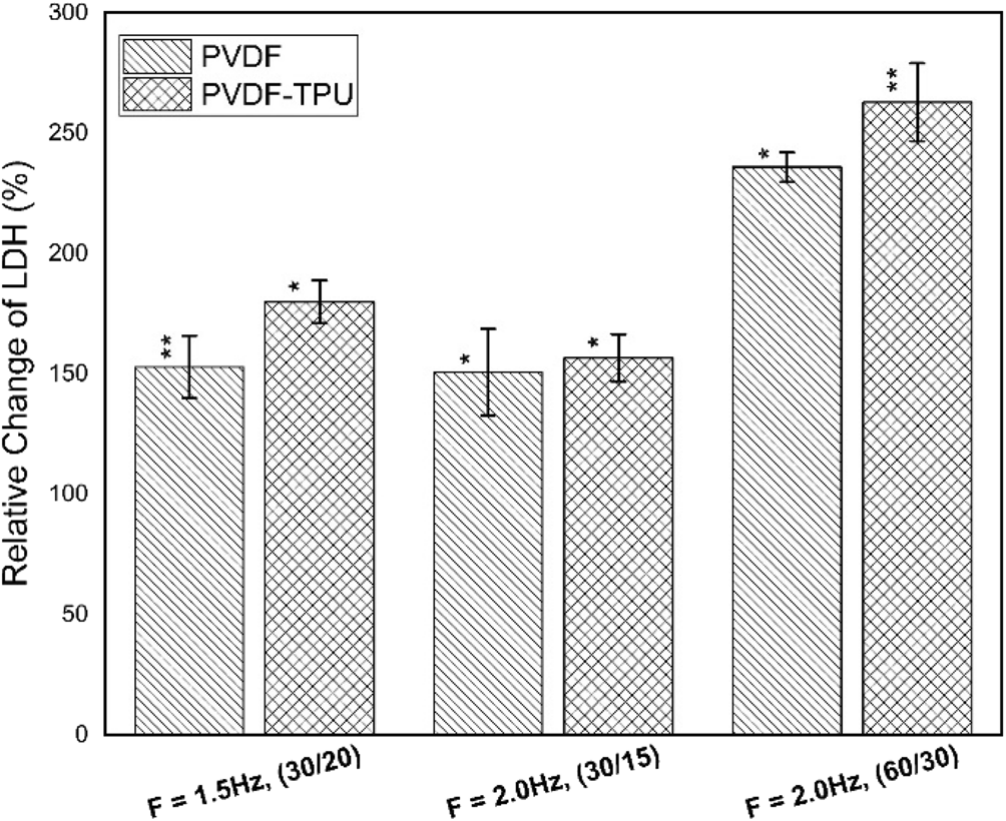
The relative change of LDH% of k. penomenue samples exposed to PEFPs generated from PVDF and PVDF-TPU nanofibers at 1.5 Hz-20 s, 2.0 Hz-15 s and 2.0 Hz-30 s.

The study was extended to include the intracellular protein leakage from bacterial membranes into the extracellular medium and then measured as percentage of leakage in relative to control samples. Figure 12 shows the relative change percentages of protein leakage due to exposure to PEPs generated from PVDF and PVDF-TPU nanofibers at 1.5 Hz-20 s, 2.0 Hz-15 s and 2.0 Hz-30 s. Here, the datum level of protein leakage was considered for control samples as zero level (0%). In particular, the histogram showed enhancement of bacterial protein leakage due to exposure in all examined samples. The level of leakage was maximum for samples exposed to PEPs generated from PVDF-TPU at 60 pulse train, compared to other counts of pulses. Apparently, the leakage of protein from the bacterial cytoplasm is confirming our previous findings that inhibitory effect is in pulse number dependency. One can say that the ability of PEPs to cause local field perturbation with other cellular bio-fields may lead to cellular disruptions and dislocation of cell membrane macromolecules. Such deteriorated effect on cell membrane may expedite leakage of protein from the bacterial cytoplasm into outside^91^. In addition, the LDH as bio-marker enzyme was studied to confirm cellular injury due to exposure to PEPs in percentages of changes relative to control samples. The reflections of loss bacterial cell membrane integrity could be monitored by LDH increase due to cell death and lysis of its inter-constituents. Figure 13 shows the relative change of LDH% due to exposure to PEPs generated from PVDF and PVDF-TPU nanofibers at 1.5 Hz-20 s, 2.0 Hz-15 s and 2.0 Hz-30 s. Significantly maximum cell injury was observed for samples exposed to PEPs generated from PVDF-TPU nanofibers at 2.0 Hz-30 s. The injury confirmed ability PEPs to cause direct effect on bacteria outer membrane. From electrical point of view, we can consider the bacterial cell as a dielectric shell shape contains biomaterials and so, direct effect of PEPs is to alter the electrical properties of the outer layer of the cell membrane^92^. On the bases of heterogeneous macro and micro structure of the cell membrane and the presence of ion binding protein it may be presumed that the electrical alterations in cell envelop leaded to lose normal charge distribution^93–95^ and cause changes in the ion concentration gradient across the membrane. The changes in ionic concentration gradient caused remarkable variations in the membrane potential and hence loss of membrane molecular constituents^96,97^.

**Figure 14:**
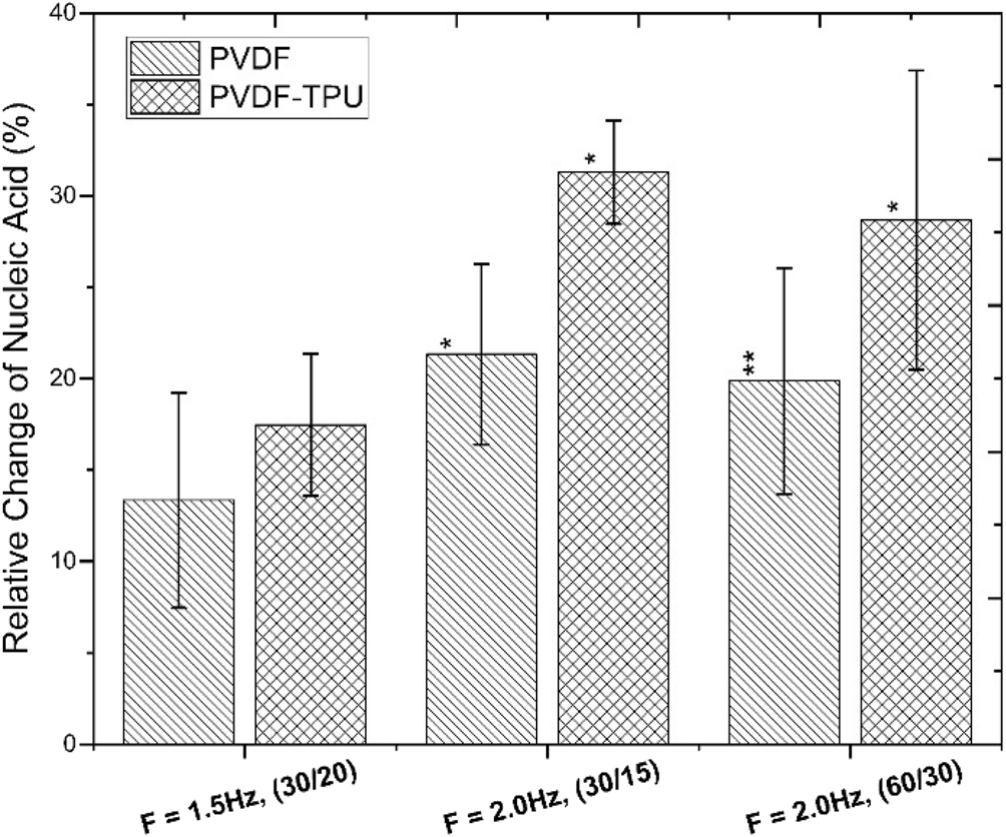
The relative change of nucleic acid percentages of k. penomenue samples exposed to PEFPs generated from PVDF and PVDF-TPU nanofibers at 1.5 Hz-20 s, 2.0 Hz-15 s and 2.0 Hz-30 s.

Moreover, to check the quality of cell inner constituents; the nucleic acid percentages were measured in relative to control samples and graphed as shown in Fig. 14. The obtained data showed inconsistent and untypical sequence of exposure effect on PEPs generated from PVDF-TPU nanofibers, where the pulses of 2.0 Hz-15 s had the maximum relative change of nucleic acid up to 30%. It may be hypothesized that the irrelevant effect in nucleic acid percentages may be resulted from the different indirect responses of inside of bacteria cells to PEPs. Here the indirect effect couldn’t be engaged to charge allocation and field disruption because the energy is not sufficient to break nucleic acid molecular bonds or DNA strands. To that end, the cell vitality is based on charge surface distribution that possess cellular intercommunication signals through transmembrane receptors allocated peripherally to the cell. Explicitly, the cell membrane structure and surface charge distribution present a conformational analysis of the possible external electric field influence^98^. The cell membrane potential resulting from charge distribution is related to cell membrane composition and gives biological characteristics of the cell^99^. Membrane stability, dynamicity and ionic uptake are dependent on the charged macromolecular head groups as key elements in modulating channels functionalized in the cell metabolic activity^100,101^. Different field perturbations are resulted from interaction of localized piezoelectric fields with charged macromolecular heads due to differences in their charge amount and bond length. The induced electric moments across membrane bilayer have reoriented and dislocated peripheral membrane macromolecules that caused intracellular metabolism to cease to exist and hence cell death.

## Conclusions

In this work, we innovatively investigated the impact of piezoelectric electrospun nanofibers and its corresponding generated voltage on the reduction of bacteria growth. This paper introduced the fabrication of both piezoelectric PVDF and PVDF: TPU nanofibers mats via electrospinning process. The piezoelectric analysis of both mats, which contain beta-sheet concentrations up to 80%, shows the generation of voltages up to 1 V under stretching strain of more than 15% on cyclic basis. Furthermore, TPU enhanced the piezoelectric response, compared to pure PVDF, under different applied mechanical excitations according to a possible better dipoles’ alignment inside the nanofibers within the added stretchable TPU element. Then, we have used both studied nanofibers to check the antibacterial impact due to cyclic stretched piezoelectricity. The experimental results show the reduction of the formed *k. penomenue* bacteria colonies due to the applied cyclic stretching, and the corresponding generated voltage of both studied nanocomposite mats. In addition, the cyclic piezoelectric performance shows a slower kinetic growth of the bacteria due to the generated electric voltage, and corresponding localized electric field exposed to bacteria. Generally, PVDF:TPU nanofibers show a slower kinetic growth of bacteria up to half the similar rate of pure PVDF nanofibers according to piezoelectric field pulses (PEPs) with a frequency up to 2 Hz. In addition, PVDF:TPU shows a relative change in the nucleic acid more than 30% and relative leakage of proteins close to 200%. In summary, The piezoelectric mechanism from both PVDF and enhanced-stretchable PVDF: TPU electrospun nanofibers positively acts as an effective anti-bacterial mats. This work is promising in applying the piezoelectricity characteristics in microbiological field within masks, filters, and wound curing applications, without depending on the traditional direct contact mechanism of anti-bacterial nanoparticles.

## Data Availability

The datasets generated and/or analyzed during the current study are not publicly available due possible patent application, but are available from the corresponding author on reasonable request.
